# Three-dimensional reconstruction of vascular arrangement including the hepatic artery and left gastric vein during gastric surgery

**DOI:** 10.1186/s40064-016-2583-9

**Published:** 2016-06-22

**Authors:** Ryoichi Miyamoto, Satoshi Inagawa, Kentaro Nagai, Michihiro Maeda, Akira Kemmochi, Masayoshi Yamamoto

**Affiliations:** Department of Gastroenterological Surgery, Tsukuba Medical Center Hospital, 1-3-1 Amakubo, Tsukuba, Ibaraki 305-8558 Japan

**Keywords:** Three-dimensional reconstruction, Gastric surgery, Laparoscopy-assisted distal gastrectomy, Left gastric vein, Hepatic artery

## Abstract

**Background:**

During gastric surgery, precise recognition of the anatomical variations and relationships among gastric tumors and vessels, including the hepatic artery (HA) and left gastric vein (LGV), is required. We utilized a three-dimensional (3D) reconstructed image as a preoperative simulation for gastric surgery.

**Methods:**

We retrospectively analyzed 84 patients who underwent gastrectomy at Tsukuba Medical Center Hospital. This cohort was sequentially divided into a without-3D group (n = 42) and with-3D group (n = 42), and the perioperative outcomes were compared. The 3D image could be used to classify the HA or LGV arrangement pattern.

**Results:**

Regarding the HA arrangement, the right HA of 1 patient (2.3 %) was arising from the superior mesenteric artery, the left HA of 8 patients (19 %) was arising from the left gastric artery, 29 patients (69 %) presented a normal rearrangement, and 4 patients (9.5 %) exhibited other arrangements. The analysis of the LGV arrangement revealed that the LGV in 15 patients (36 %) was located on the dorsal side of the common HA, the LGV in 5 patients (12 %) was located on the ventral side of the common HA, the LGV in 12 patients (29 %) was found on the ventral side of the splenic artery, the LGV in 6 patients (14 %) was located on the dorsal side of the splenic artery, and 4 patients (9.5 %) presented other arrangements. The intraoperative blood loss in the without-3D and with-3D groups was 276 ± 430 and 157 ± 170 g, respectively (*p* = 0.027).

**Conclusions:**

The 3D reconstruction technique was useful for understanding and sharing anatomic information during gastric surgery.

## Background

During gastric surgery, local lymph node excision commonly involves excision of the perigastric lymph nodes, such as the lymph nodes around the left gastric artery trunk (classification 7), the lymph nodes in the anterosuperior region of the common hepatic artery (classification 8a), and the lymph nodes around the celiac artery (classification 9), according to the Japanese classification of gastric carcinoma (Japanese Gastric Cancer Association 1998). Specifically, the areas near the left gastric vein (LGV) and hepatic artery (HA), which are known to contain various anatomical variations, are challenging sites for radical lymph node dissection. Therefore, preoperative assessment of the precise arrangement of the perigastric vascular supply, including the LGV and HA, might avoid unnecessary bleeding and facilitate a safe, rapid gastric surgery (Huang et al. 2014; Rebibo et al. 2012).

As for the vascular components, including the HA and LGV, Jonathan et al. and Koops et al. previously reported that an unusual HA arrangement is present in 21–25 % of patients undergoing hepatic surgery (according to angiography) (Koops et al. 2004; Hiatt et al. 1994). Kawasaki et al. (2010) reported that the LGV arrangement pattern could be classified into five groups by 3DCT angiography. Similarly, Sakaguchi et al. (2010) reported a confluent pattern of the LGV to the portal vein or splenic vein.

However, the above-mentioned 3D reconstruction techniques provide only an anatomical mapping of the blood vessel arrangement. Therefore, it has been very difficult to appreciate the precise anatomical variations and relationships among gastric tumors, vessels, and surrounding organs. To solve this problem, we developed a novel type of 3D surgical reconstruction imaging technique created from MDCT images. We utilized this technique for preoperative simulation and intraoperative navigation during gastric surgery, including laparoscopy-assisted distal gastrectomy.

Here, we examined the HA and LGV arrangements in patients undergoing gastric surgery with a preoperative 3D reconstruction imaging technique. Furthermore, we also evaluated the extent to which preoperative 3D reconstruction affected the perioperative outcomes of gastric surgery.

## Methods

### Patient characteristics and perioperative outcomes

We retrospectively analyzed 84 patients who underwent gastric surgery at Tsukuba Medical Center Hospital between April 2014 and December 2015. Because we have only used 3D surgical simulations since January 2015, this cohort could be sequentially divided into a group that did not undergo 3D surgical simulation (n = 42) and a group in which 3D surgical simulation was used (n = 42). The ethics committee of the Tsukuba Medical Center Hospital approved this study. We included patients who underwent standard surgery with gastric cancer systemic lymph node dissection and excluded patients with severe tumor stenosis and those with possible invasion of other organs by primary tumors. The patient characteristics, i.e., age, sex, body mass index (BMI), American Society of Anesthesiology (ASA) score, performance status, histopathological stage, surgical procedure, type of surgeon (a surgical resident who was in their fifth to fourteenth postgraduate year or a senior gastric surgeon), perioperative outcomes (i.e., operating time, intraoperative blood loss, and length of postoperative hospital stay), and postoperative complications were compared between the two groups. The postoperative complications were graded according to Clavien’s classification (Dindo et al. 2004). Furthermore, some previous studies reported that obesity is strongly related to perioperative outcomes in gastric surgery (Makino et al. 2008; Inagawa et al. 2000). According to the guidelines of the World Health Organization (2000), we divided our without-3D and with-3D groups into two subgroups: those with a low BMI (BMI < 25 kg/m^2^, n = 40) and those with a high BMI (BMI ≥ 25 kg/m^2^, n = 44). The perioperative outcomes of the patients were compared between the two groups.

## 3D images used in the present study

We used a workstation (AZE Ltd, Virtual Place Advance 300, Tokyo, Japan) to construct 3D images from MDCT images. This software offers a standardized analysis of 3D anatomy based on two-dimensional MDCT imaging with contrast media. Furthermore, gastric cancer patients were administered a vesicant to expand their stomach during the routine preoperative MDCT imaging. This approach revealed gastric wall tumors and the perigastric vascular arrangement in a 3D reconstructed image. A preoperative conference based on the 3D images enabled the 3D anatomic images to be shared with the surgical staff. Furthermore, the surgical team was able to view the preoperative 3D image on a large display during the actual surgery, enabling discussion of the critical points of the surgical procedure.

### Classification of HA and LGV arrangement using 3D imaging

**Fig. 1:**
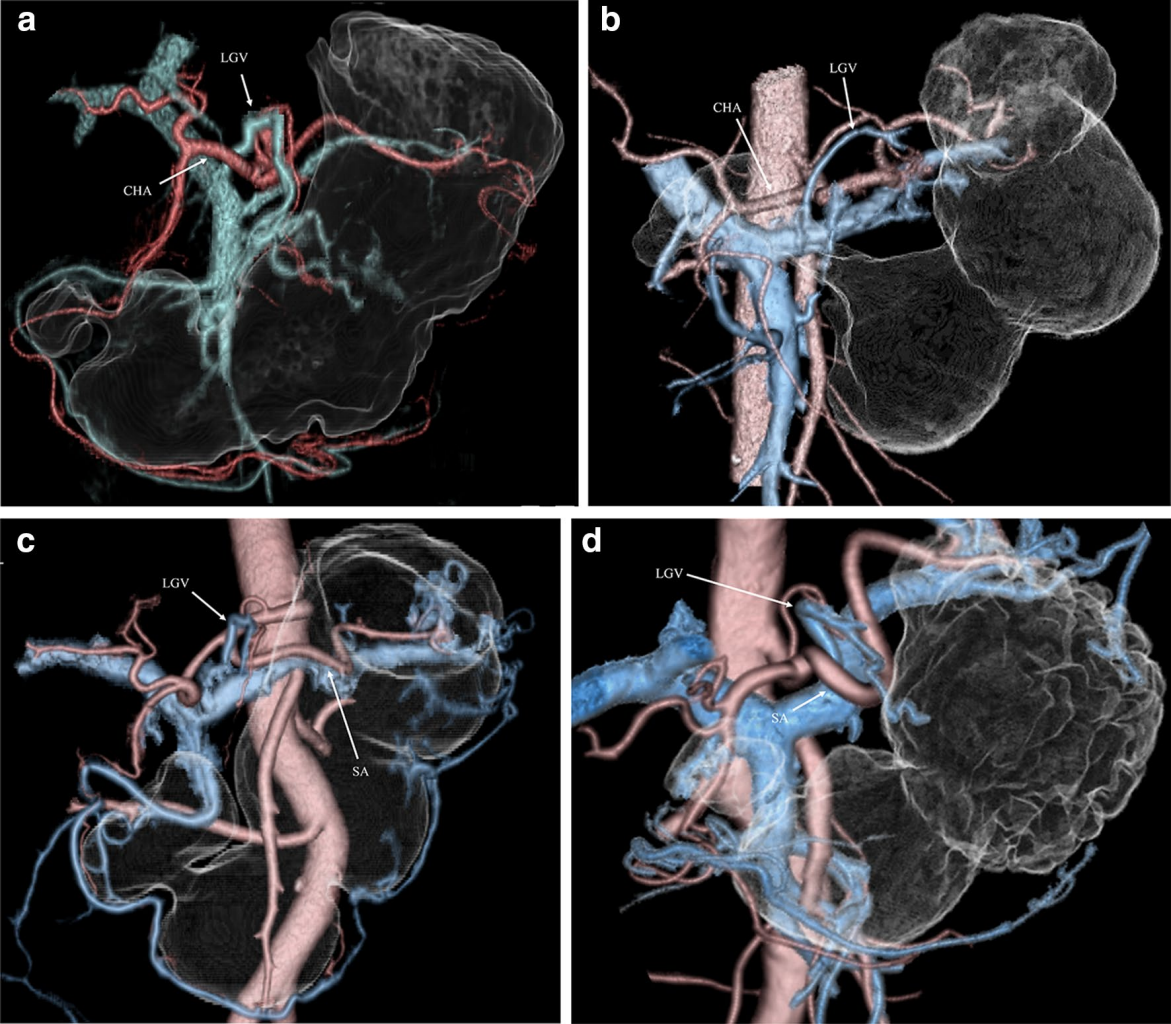
Reconstructed 3D images of the perigastric vessels, including the left gastric vein (LGV) and gastric tumor, in a patient with gastric cancer are shown. The course of the LGV was divided as follows. The images in **b** and **c** were respectively obtained from the same patient. **a** The dorsal side of the common hepatic artery (type A). **b** The ventral side of the common hepatic artery (type B). **c** The ventral side of the splenic artery (type C). **d** The dorsal side of the splenic artery (type D). *LGV* left gastric vein, *CHA* common hepatic artery, *SA* splenic artery

Among the 42 patients who underwent 3D surgical simulation, we evaluated the vessel arrangements, including the HA and LGV. We divided the course of the hepatic artery into four groups—i.e., type I: the (accessory) right HA arising from the superior mesenteric artery; type II: the (accessory) left HA arising from the left gastric artery; type III: the most common pattern; and type IV: other rearrangements. Regarding LGV arrangements, we categorized the branching pattern of the LGV into five groups according to a previous report, i.e., type A: in the dorsal side of the common hepatic artery (CHA) (Fig. 1a); type B: in the ventral side of the CHA (Fig. 1b); type C: in the ventral side of the splenic artery (SA) (Fig. 1c); type D: in the dorsal side of the SA (Fig. 1d), and type E: other patterns (Kawasaki et al. 2010).

### Statistical analyses

The correlation of the two groups was analyzed using the χ^2^ test or Fisher’s exact test as appropriate. Statistical analyses were performed using a statistical analysis software package (Version 21; IBM, Armonk, NY), and *p* values <0.05 were considered significant.

## Results

### Patient characteristics

There were no significant differences regarding patient characteristics between the without-3D and with-3D groups (Table 1).

**Table 1:** Patient characteristics

Factors	Without-3D (n = 42)	With-3D (n = 42)	*p* value
Age	71 (31–97)	71 (46–94)	0.310
Sex ratio (male:female)	32:10	26:16	0.142
BMI (kg/m^2^)	23.3 ± 3.25	23.2 ± 3.38	0.535
ASA score (1–4)	2.05 ± 0.59	2.00 ± 0.61	0.556
Performance status (0–4)	0.21 ± 0.51	0.13 ± 0.45	0.718
*Stage (UICC 7th)*			
IA, IB	18	16	0.479
IIA, IIB, IIC	3	12	
IIIA, IIIB, IIIC	11	12	
IVA, IVB	10	2	
*Surgical procedure*			
LADG	4	7	0.257
Distal gastrectomy	16	18	
Proximal gastrectomy	2	2	
Total gastrectomy	20	15	
*Type of surgeon*			
Surgical resident	28	29	0.815
Senior surgeon	14	13	

*3D* three-dimensional, *BMI* body mass index, *ASA* American Society of Anesthesiology, *UICC* Union for International Cancer Control, *LADG* laparoscopy-assisted distal gastrectomy

*The HA and LGV vessel arrangements as classified by 3D imaging* (Table 2).

**Table 2:** Classification of the HA and LGV

Type of the vessels arrangement	n = 42
*The HA arrangement*	
Type I	1 (2.3 %)
Type II	8 (19 %)
Type III	29 (69 %)
Type IV	4 (9.5 %)
*The LGV arrangement*	
Type A	15 (36 %)
Type B	5 (12 %)
Type C	12 (29 %)
Type D	6 (14 %)
Type E	4 (9.5 %)

*HA* hepatic artery, *LGV* left gastric vein

Regarding the HA arrangements according to the 3D images, 1 patient (2.3 %) was type I, 8 patients (19 %) were type II, 29 patients (69 %) were normal, and 4 patients (9.5 %) were type IV (other). As for the branching type of the LGV according to the 3D images, 15 patients (36 %) were type A, 5 patients (12 %) were type B, 12 patients (29 %) were type C, 6 patients (14 %) were type C, and 4 patients (9.5 %) were type E (other).

### Perioperative outcomes

Intraoperative blood loss was significantly reduced in the with-3D group (157 ± 170 g) compared to the without-3D group (276 ± 430 g) (*p* = 0.027). However, a comparison of perioperative outcomes between the two groups did not reveal significant differences in the operating time, length of postoperative hospital stay, or Grade III–V postoperative complications (Table 3).

**Table 3:** Perioperative outcomes

Factors	Without-3D (n = 42)	With-3D (n = 42)	*p* value
Postoperative complications (≧Clavien grade III)	3 (7.1 %)	4 (9.5 %)	0.350
Operating time (minutes)	250 ± 55	264 ± 60	0.216
Intraoperative blood loss (g)	276 ± 430	157 ± 170	0.027
Length of postoperative hospital stay (days)	11 (7–49)	11 (8–36)	0.535

*3D* three-dimensional

### Correlating effect between patients’ obesity and 3D imaging on perioperative outcomes

There were no significant differences in the patient characteristics between the low-BMI and high-BMI groups (data not shown). In the high-BMI groups, a significant difference in intraoperative blood loss was observed between the with-3D (156 ± 135 g) and without-3D groups (351 ± 566 g) (*p* = 0.001). However, in the low-BMI groups, no significant difference in intraoperative blood loss was observed between the with-3D (123 ± 111 g) and without-3D groups (139 ± 115 g) (*p* = 0.153).

In terms of other perioperative outcomes, including the operating time, length of postoperative hospital stay, and Grade III–V postoperative complications, significant differences were not observed between the with-3D and without-3D groups (Table 4).

**Table 4:** BMI depended perioperative analysis

Factors	Low-BMI (n = 40)		*p* value	High-BMI (n = 44)		*p* value
	Without-3D (n = 20)	With-3D (n = 20)		Without-3D (n = 22)	With-3D (n = 22)	
Postoperative complications (≧Clavien grade III)	1	2	0.556	2	2	1.000
Operating time (minutes)	234 ± 49	254 ± 73	0.213	265 ± 58	271 ± 42	0.456
Intraoperative blood loss (g)	139 ± 115	123 ± 111	0.153	351 ± 566	156 ± 135	0.001
Length of postoperative hospital stay (days)	11 (7–45)	11 (7–35)	0.435	11 (7–49)	11 (9–40)	0.471

*BMI* body mass index, *3D* three-dimensional

## Discussion

Preoperative anatomical 3D imaging facilitated the classification of the HA and LGV branching patterns and revealed the relative positions of the gastric tumors, HA, and LGV during gastric surgery. These findings were helpful and allowed the anatomical images to be shared with surgical staff to promote a safe gastric surgery.

In 1994, laparoscopy-assisted surgery was developed for the treatment of gastric cancer in Japan. Laparoscopy-assisted surgery is an attractive approach due to its capacity to improve the quality of life of people with gastric cancer (Kitano et al. 1994; Azagra et al. 1999; Uyama et al. 1999; Matsuki et al. 2004). Laparoscopy-assisted distal gastrectomy is less invasive than open surgery. However, one disadvantage of laparoscopy-assisted distal gastrectomy is that it is difficult to obtain an anatomical image of the entire lesion. In addition, using this approach, we could not manipulate the lesion directly. For these reasons, a relatively long intraoperative time is required to identify the origins of arteries and veins, and the specific anatomy of the vasculature varies greatly from case to case (Azagra et al. 1999). To solve these problems, we applied 3D imaging during gastric surgery, including laparoscopy-assisted distal gastrectomy. The reconstructed 3D image could be easily observed from any angle. If the direction of the laparoscope observing abdominal organs is predicted, a 3D image can be reconstructed as the laparoscope’s field of view (Takiguchi et al. 2004; Uyama et al. 1999).

When systemic regional lymphadenectomy (classification 12a) in the hepatoduodenal ligament is performed, the hepatic artery should be precisely identified. Referring to our classification, the 1000 patients in Jonathan’s study could be reclassified as 106 (10 %) type I, 97 (9.7 %) type II, 757 (75 %) normal type and 38 (3.8 %) other patients (Hiatt et al. 1994). Similarly, the 604 patients in Koops’ study could be reclassified as 72 (12 %) type I, 26 (4.3 %) type II, 477 (79 %) type III and 29 (4.8 %) other patients (Koops et al. 2004). These previous reports classified the hepatic artery pattern using angiography. The present study classified hepatic artery patterns as our original four types without using angiography. We assumed that our reconstructed 3D imaging technique is less invasive for patients.

Considering that major bleeding occurs mainly from the laceration of fragile veins, more emphasis should be placed on the precise recognition of LGV anatomy. In Sakaguchi et al. (2010), the LGV confluence pattern was portal vein in 37 patients (40 %) and splenic vein in 44 (48 %). Kawasaki et al. (2010), utilizing 3DCT angiography, reported that the most frequent location of the LGV was dorsal to the common hepatic artery. Similar to these previous reports, the present study clearly classified the confluent pattern of the LGV around the stomach using our 3D reconstructed images.

Although there have been several studies regarding venous anatomy of the LGV (Douglass et al. 1950; Hiwatashi et al. 1999; Roi 1993), the reasons for the different drainage patterns remain unclear. Embryologically (Huang et al. 2014), the primitive foregut venous plexus (PFVP) courses along the primitive foregut, while the ductus venosus (DV of Arantius) is anastomosed to the PFVP resulting in anastomotic omental veins (AOVs). During standard development of the PFVP, the AOVs gradually disappear, and both the right and left gastric veins end in the main PV. The veins, however, must adapt to changes in the intestinal canal and expansion of the liver (resulting in different drainage).

Significantly less intraoperative blood loss was observed in the with-3D group compared to the without-3D group. Thus, an improved preoperative understanding of the 3D anatomy, including the vessel and gastric tumor arrangement, and sharing the 3D images with the surgical staff was assumed to contribute to a safe gastric surgery (during laparoscopy-assisted distal gastrectomy) and a mastery of the surgical techniques and instruments and perioperative management. In particular, our 3D reconstruction technique was hypothesized to be useful for highly obese patient. A strong relationship between obesity and intraoperative blood loss in patients has been widely discussed (Makino et al. 2008; Inagawa et al. 2000). When operating, we found it more difficult to grasp the surgical anatomy of a highly obese patient than that of a patient with a low degree of obesity. Furthermore, high-fat tissue can be easily lacerated during surgery. These disadvantages are hypothesized to result in more intraoperative bleeding during the operation of a highly obese patient. We assumed that our 3D reconstruction technique allow us to grasp the precise 3D anatomy of a highly obese patient, including the vessel and gastric tumor arrangement, during surgery. In fact, the present study indicates that the intraoperative blood loss of highly obese patients is significantly reduced in the with-3D group (Table 4).

This novel modality has several limitations that must be addressed. First, a considerable amount of time (approximately 30 min to 1 h at present) was required to create the detailed reconstructed 3D images because of the performance of the software system. Second, our reconstructed 3D images had a tendency to misclassify superficial flat and depressed lesions, especially those located in the gastric cardia and antrum, because of insufficient expansion of the stomach wall. Third, this investigation was a retrospective study with a relatively small number of patients at a single institution. In fact, the with-3D group is likely to have undergone more laparoscopic surgeries and less open total gastrectomies, and include more stage II and less stage IV patients compared with the without-3D group. Therefore, we assumed that the disease stage and surgical procedure may influence our results. Hence, these results will need to be confirmed through additional large-scale studies.

## Conclusion

The present 3D reconstruction technique was useful for determining the relative positions of vessels, including the HA and LGV, and the gastric tumor, during gastric surgery, particularly in highly obese patients. We propose the use of this 3D reconstruction technique as a novel modality for preoperative assessment and intraoperative navigation during gastric surgeries, including laparoscopy-assisted distal gastrectomy.
